# The combination of immune checkpoint inhibitors and antibody-drug conjugates in the treatment of urogenital tumors: a review insights from phase 2 and 3 studies

**DOI:** 10.1038/s41419-024-06837-w

**Published:** 2024-06-19

**Authors:** Puguang Yu, Chunming Zhu, Xiangyun You, Wen Gu, Xia Wang, Yuan Wang, Renge Bu, Kefeng Wang

**Affiliations:** 1 Department of Urology, Shengjing Hospital of China Medical University, Shenyang, 110004 China; 2 Department of Family Medicine, Shengjing Hospital of China Medical University, Shenyang, 110004 China; 3 Department of Urology, The First College of Clinical Medical Science, China Three Gorges University, Yichang, 443002 China; 4 Department of Urology, Yichang Central People’s Hospital, Yichang, 443002 China; 5 Department of Oncology, Shengjing Hospital of China Medical University, Shenyang, 110004 China; 6 Department of General Surgery, Shengjing Hospital of China Medical University, Shenyang, 110004 China

**Keywords:** Drug development, Urological cancer, Immunotherapy

## Abstract

With the high incidence of urogenital tumors worldwide, urinary system tumors are among the top 10 most common tumors in men, with prostate cancer ranking first and bladder cancer fourth. Patients with resistant urogenital tumors often have poor prognosis. In recent years, researchers have discovered numerous specific cancer antigens, which has led to the development of several new anti-cancer drugs. Using protein analysis techniques, researchers developed immune checkpoint inhibitors (ICIs) and antibody-conjugated drugs (ADCs) for the treatment of advanced urogenital tumors. However, tumor resistance often leads to the failure of monotherapy. Therefore, clinical trials of the combination of ICIs and ADCs have been carried out in numerous centers around the world. This article reviewed phase 2 and 3 clinical studies of ICIs, ADCs, and their combination in the treatment of urogenital tumors to highlight safe and effective methods for selecting individualized therapeutic strategies for patients. ICIs activate the immune system, whereas ADCs link monoclonal antibodies to toxins, which can achieve a synergistic effect when the two drugs are combined. This synergistic effect provides multiple advantages for the treatment of urogenital tumors.

## Facts

- ICIs can alleviate immunosuppression in the tumor microenvironment and stimulate the body’s autoimmune response, while ADCs can combine the targeting ability of monoclonal antibodies with the cytotoxicity of anti-tumor chemotherapy drugs through specific biochemical linkers.
- ICIs combined with ADCs can produce synergistic effects in the treatment of urogenital tumors.
- Phase 2 and 3 clinical trials of ADCs, ICIs, and their combined therapy may provide new suggestions for the clinical treatment of urogenital tumors.

## Open questions

- How to improve the highly selectivity and cytotoxicity of ICIs to urogenital tumor cells without harming normal tissues?
- How to avoid or delay the occurrence of hyper-progressive diseases in the use of ICIs in urogenital tumors?
- How to systematically evaluate the physical status, hematological indicators, and target organs function of urogenital tumors patients using ADCs to reduce the occurrence of adverse reactions?
- How to optimize the administration sequence and dose selection of combination therapy and select reliable biomarkers to predict the anti-tumor effects in urogenital tumors?

## Introduction

According to a global cancer statistics report, there is a high incidence of urogenital tumors worldwide [1]. Among men, urinary system tumors are among the top 10 most common tumors, with prostate cancer (PCa) ranking first and bladder cancer (BC) fourth [2]. At present, the main treatment for early urogenital tumors is surgical resection, whereas advanced patients are usually treated with chemotherapy, radiotherapy, immunotherapy, or targeted therapy to improve patients’ quality of life [3]. Unfortunately, patients with resistant tumors often have a poor prognosis [4], presenting an urgent need for new clinical trials to determine more effective treatment strategies for advanced patients.

The immune system plays a crucial role in the occurrence and development of tumors and therefore has an important impact on patient prognosis [5]. To restore the body’s natural anti-tumor-immune response, tumor immunotherapy can be employed as a therapeutic approach to control and eliminate tumors by reactivating and maintaining the tumor-immune cycle [6]. In recent years, significant advancements have been made in the development and research of immune checkpoints and related inhibitors such as cytotoxic T-lymphocyte-associated protein-4 (CTLA-4), programmed death protein-1 (PD-1), and programmed death ligand-1 (PD-L1). These advancements have shown promising prospects for immunotherapy, heralding a new era in cancer treatment [7].

Although immune checkpoint inhibitors (ICIs) have the advantages of targeted therapy, fewer adverse events (AEs), and improved efficacy, they impose certain limitations in some individuals, such as limited reaction, toxicity, and drug resistance [8]. Currently, research on immunotherapy drugs is challenged by the need to select other treatment regimens to use in combination with immunotherapy drugs so as to mitigate the occurrence of immunotherapy-related AEs, which involves accurately determining the efficacy of the immunotherapy, identifying suitable and contraindicated individuals for treatment, and improving the therapeutic efficacy for genetically mutated tumor cells [9]. Due to the immune microenvironment (IME) of urogenital tumors, few patients benefit from ICI treatment [10]. For instance, PCa is a typical “cold” tumor with a very low response rate to ICI treatment [11]. Future research focusing on transforming “cold” tumor into “hot” tumor may enhance the response to ICIs.

Antibody drug conjugates (ADCs) represent an important advancement in cancer treatment, following precision targeted therapy and immunotherapy. Several ADCs, including enfortumab vedotin (EV), sacituzumab govitecan (SG), and oportuzumab monatox (OM), have been used for the treatment of urothelial carcinoma (UC), both domestically and internationally [12]. With the emergence of technologies that enable personalized diagnosis and treatment, and the advent of precision medicine, ADCs have become a promising therapeutic strategy for urogenital tumors. They offer hope to patients, especially those who have previously failed immunotherapy. With their efficacy and safety, ADCs are expected to play a significant role in the treatment of urogenital tumors.

**Fig. 1:**
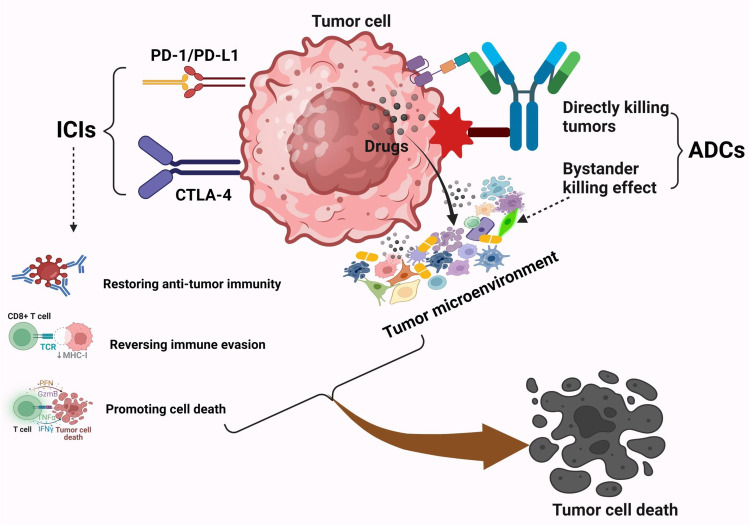
ICIs combined with ADCs can produce synergistic effects in the treatment of urogenital tumors. ICIs (PD-1/PD-L1 and CTLA-4) inhibitors play a crucial role in activating the body’s natural anti-tumor-immune response by restoring anti-tumor immunity, reversing immune evasion, and promoting cell death pathways of tumor cells. There are two mechanisms by which ADCs eliminate tumors: (1) The first mechanism is to use the antibody component of ADCs to target tumor-specific antigens and release small-molecule cytotoxic drugs that directly kill tumor cells. (2) The second mechanism involves inducing the bystander effect of ADCs. The two mechanisms synergistically affect the TME, leading to tumor cell death. Created with BioRender.com.

Remarkable progress has been made in recent years in the treatment of urogenital tumors by using the combination of ADCs and ICIs [13]. ADCs and ICIs possess complementary mechanisms of action that provide different therapeutic effects [14]. As shown in Fig. 1, ICIs as a monotherapy enhance the immune system’s ability to kill tumor cells [10], while ADCs as a monotherapy directly target and eliminate tumor cells [15]. The combined use of ADCs and ICIs has a synergistic effect, which can enhance the therapeutic effects of each [16].

**Fig. 2:**
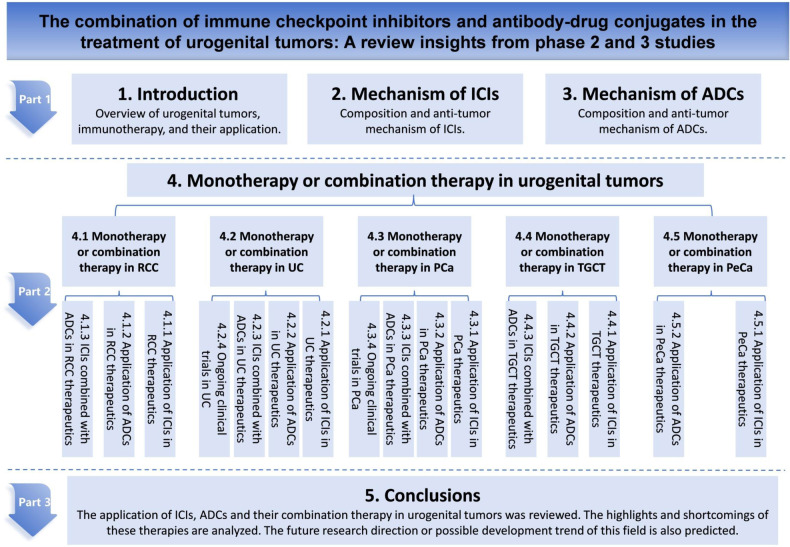
An overview of this review.

Because of the potential of this combination therapy to improve the prognosis for patients with advanced urogenital tumors that are resistant to monotherapy, the purpose of this review was to investigate the effects, clinical efficacy, and safety of ADCs combined with ICIs in phase 2/3 clinical trials of this combination therapy in the treatment of urogenital tumors, and explore its potential applications. An overview of this review, shown in Fig. 2, will guide readers in navigating the extensive information.

## Mechanism of ICIs

Immune checkpoints comprise a group of molecules that play a crucial role in regulating the body’s immune response, during which they maintain immune tolerance and minimize damage to tissues [17]. The combination of CTLA-4 and CD80/CD86 inhibits the activation of T cells and plays a negative role in immune response regulation [18]. Regulatory T cells (Tregs) can reduce the expression of CD80/CD86 through CTLA-4-dependent cytosis, which leads to reduced stimulatory activity of antigen-presenting cells on T cells [19]. Additionally, Tregs increase the activity of PD-1 and PD-L1 on effector T cells, resulting in dual inhibition of T cells [20]. PD-L1 is primarily expressed on tumor cells, and interaction with PD-L1 mainly occurs in the tumor microenvironment (TME) [21]. Therefore, PD-1 and PD-L1 inhibitors are associated with fewer autoimmune side effects. Lymphocyte activating gene-3 is expressed on Tregs and inactivated CD4+Th cells, and binds to major histocompatibility complex class II molecules, thereby inhibiting CD4 + T-cell activity [22].

## Mechanism of ADCs

ADCs are composed of monoclonal antibodies that target specific antigens, small-molecule cytotoxic drugs, and linkers [23]. They combine the cancer cell killing effect of traditional small-molecule chemotherapy with the tumor-targeting ability of antibody drugs [24]. When ADCs are administered intravenously, they recognize and bind to tumor surface antigens. As the antigen undergoes endocytosis, the ADC enters the cancer cells through the endocytic pathway, where they are then transported to lysosomes and degraded until the payload is released in a biologically active form, leading to the death of the cancer cells [25]. ADCs exert antitumor activity through either of two main pathways. In the first pathway, specific antibodies bind to cell surface antigens, are internalized by tumor cells via the endolysosomal system, and release payloads into the cytoplasm, inducing cytotoxicity [26]. The second pathway induces apoptosis and tumor cell death via bystander killing [27].

## Monotherapy or combination therapy in urogenital tumors

Urogenital cancers encompass various types of tumors affecting the urogenital system, including BC, renal cell carcinoma (RCC), PCa, testicular germ cell tumor (TGCT), and penile cancer (PeCa). Treatment approaches consist of either monotherapy or combination therapy.

### Monotherapy or combination therapy in RCC

The incidence of RCC accounts for approximately 2%–3% of adult malignant tumors, ranking ninth among male tumors worldwide [28]. It is the second most common malignant tumor in the urinary system, after PCa and BC [28]. At present, the treatment of advanced RCC (aRCC) has evolved from traditional radiotherapy and chemotherapy to targeted therapy and immunotherapy [29]. Clinical trials of ICIs (such as PD-1/PD-L1 or CTLA-4) have revealed the limited efficacy of this monotherapy [30]. Meanwhile, ADCs in combination with immunotherapy and other treatments have shown the potential to improve patient prognosis, and have thus emerged as a major prospective therapeutic pathway for aRCC in the future [31].

#### Application of ICIs in RCC therapeutics

The IME of RCC is characterized by a high frequency of somatic mutations and tumor-infiltrating lymphocytes [32]. In 2021, Krishna et al. [33] conducted single-cell transcriptome sequencing of 167,283 cells from six patients (two with immune checkpoint blockade [ICB]-naïve and four with multiple tumor regions, lymph nodes, normal kidneys, and peripheral blood treated with ICB). T-cell receptor (TCR) sequencing was performed to comprehensively analyze the IME in completely resected high-risk clear cell RCC (ccRCC). The study indicated widespread heterogeneity in the IME and TCR across different patients and samples, highlighting the complexity of the immune response in RCC and revealing potential targets for immunotherapeutic drugs.

##### Anti-PD-1 inhibitors

Showing promising results, nivolumab is the first PD-1 monoclonal antibody to be approved for the treatment of aRCC. As shown in Table 1, the CheckMate 025 study confirmed that nivolumab was superior to everolimus in short- and long-term efficacy, safety, and tolerability in aRCC patients taking nivolumab 3 mg/kg every 2 weeks or everolimus 10 mg daily. From the perspective of short-term overall survival (OS) and objective response rate (ORR), nivolumab benefitted patients in multiple subgroups. At the same time, it presented no obvious safety concerns, further supporting nivolumab as the standard treatment for previously treated aRCC patients [34]. In the long-term trial, the superior efficacy of nivolumab over everolimus (OS: 25.8 months vs. 19.7 months, respectively; nivolumab, higher ORR) was maintained after extended follow-up, with no new safety signals, thus supporting the long-term benefits of nivolumab monotherapy in patients with previously treated aRCC [35].

**Table 1:** Clinical trials of ICIs, ADCs, and their combined application in RCC.

Categories	Clinical trials	Phases	Patients	Drugs	Targets	Clinical outcomes	References
ICIs	CheckMate 025	3	821 aRCC	Nivo	PD-1	Nivo demonstrated an OS improvement vs. Ever; ORR also favored in Nivo.	[34]
	CheckMate 025 (Long-term)	3	821 aRCC	Nivo	PD-1	OS: 25.8 m vs. 19.7 m; 5-year OS: 26% vs. 18%; ORR: 23% vs. 4%; PFS also favored in Nivo.	[35]
	CLEAR	3	1069 aRCC	Pemb + Lenv	PD-1 + TKIs	PFS: Pemb+Lenv vs. Suni: 23.9 m vs. 9.2 m; Lenv + Ever vs. Suni: 14.7 m vs. 9.2 m.	[37]
	Checkmate 9ER	3	1003 a/mRCC	Nivo + Cabo/Suni	PD-1 + TKIs	mOS: 37.7 m vs. 34.3 m; mPFS: 16.6 m vs. 8.3 m.	[38]
	Keynote-426	3	861 a/mRCC	Pemb + Axit	PD-1 + TKIs	ORR: 59.3% vs. 35.7%; mPFS: 15.1 m vs. 11.1 m.	[39]
	Checkmate 214	3	1096 aRCC	Nivo + Ipil	PD-1 + CTLA-4	OS: ITT (0.59 to 0.81) and I/P (0.54 to 0.78); ORR: ITT (39.1% vs. 32.4%) and I/P (41.9% vs. 26.8%).	[40]
	COSMIC-313	3	855 RCC	Nivo + Ipil + Cabo	PD-1 + CTLA-4 + TKIs	1 year PFS in experimental group vs. control group: 0.57 vs. 0.49.	[41]
ADCs	NCT02639182	2	133 mRCC	AGS-16C3F	ENPP3	mPFS: 2.9 m vs. 5.7 m; OS: 13.1 m vs.15.4 m.	[44]
	IMMU-132-01	2	515 cancers (including RCC)	SG	Trop-2	The clinical benefit rate: 45.4%; mPFS: 5.5 m; OS: 13.0 m.	[46]

##### Anti-PD-1 inhibitors plus anti-TKI inhibitors

Drug resistance indicates limited efficacy of a single-agent first-line immunotherapy, suggesting that combined therapy is needed to improve efficacy. Tyrosine kinase inhibitors (TKIs) have emerged as common targets for targeted treatment of RCC, such as cabozantinib, a TKI that targets multiple receptor tyrosine kinases involved in tumor growth, angiogenesis, metastasis, and immune regulation [36]. Therefore, the combination of ICIs and TKIs emerged as a promising therapeutic strategy for treating RCC, becoming a model strategy for combination treatment.

As shown in Table 1, the CLEAR trial [37] (pembrolizumab + lenvatinib) and the CheckMate 9ER trial [38] (nivolumab + cabozantinib/sunitinib) demonstrated that ICI/VEGFR-TKI combination therapy was superior to sunitinib monotherapy. The CLEAR trial found that median progression-free survival (mPFS) was 23.3 months in the pembrolizumab + lenvatinib group and 9.2 months in the sunitinib group, with a median OS (mOS) follow-up of 33.7 months and 33.4 months, respectively. Similarly, in the CheckMate 9ER trial, nivolumab + cabozantinib demonstrated better efficacy than sunitinib through extended follow-up and preplanned final OS analysis for each regimen. These results supported the use of PD-1 inhibitors + TKI inhibitors as the first-line therapy for aRCC patients.

Furthermore, another TKI inhibitor, axitinib, also significantly improved OS, PFS, and ORR in previously untreated aRCC/metastatic RCC (a/mRCC) patients administrated pembrolizumab + axitinib, while having a controllable safety profile. As shown in Table 1, the Keynote-426 study enrolled 861 patients with previously untreated aRCC who received pembrolizumabs + axitinib or sunitinib to compare whether pembrolizumab + axitinib produced better outcomes than sunitinib. As expected, the ORR was 59.3% in the pembrolizumab + axitinib group and 35.7% in the sunitinib group, with a mPFS of 15.1 months and 11.1 months, respectively [39].

These important findings established the foundation for using ICIs in combination with TKI inhibitors for the treatment of RCC.

##### Anti-PD-1 inhibitors plus anti-CTLA-4 inhibitors

In addition to studying the combined therapy of ICIs and TKIs, the researchers also investigated the combination of two ICIs, and the triple combination of two ICIs and a TKI. The CheckMate 214 trial [40] showed that two ICIs (a combination of PD-1 and CTLA-4) provided significant clinical benefits compared to VEGFR inhibitors (sunitinib) in patients with previously untreated aRCC. Nivolumab combined with ipilimumab not only prolonged OS, 4-year PFS, and ORR, but demonstrated commendable safety. A recent study (COSMIC-313) published in the *New England Journal of Medicine* evaluated the efficacy and safety of nivolumab in combination with ipilimumab and cabozantinib in 855 previously untreated RCC patients at risk for moderate or poor prognosis [41]. The results showed that the PFS in previously untreated RCC patients in the nivolumab + ipilimumab + cabozantinib group was significantly higher than that in the nivolumab + ipilimumab + placebo group. However, the safety of multi-drug combination is still worth our vigilance, especially in some elderly patients with poor liver and kidney function.

These clinical trials demonstrated the growing importance of ICIs in aRCC. Only through multidisciplinary collaboration can patients with RCC achieve a better prognosis. However, considering the safety and tolerability of individual patients, serious AEs in some combination therapy patients should be critically considered by clinical decision-makers. If grade 4 or recurrent grade 3 AEs occur, and grade 2 or 3 AEs continue to occur after an adjustment of treatment, the drugs that may cause AEs should be fully analyzed, and the medication regimen should be adjusted if necessary.

#### Application of ADCs in RCC therapeutics

With the discovery of more renal cancer-specific antigens, ADCs targeting these antigens are also being developed. RCC targets that have been developed include ectonucleotide pyrophosphatase/phosphodiesterase 3 (ENPP3) and trophoblast cell surface antigen-2 (Trop-2), among others. They are important in the ADC treatment of RCC.

##### Anti-ENPP3 ADCs

ENPP family members play a significant role in various physiological activities, including nucleotide signaling and immune cell infiltration in tumors [42]. ENPP3, a hydrolase responsible for metabolizing extracellular nucleotides, is predominantly expressed in RCC [43]. AGS-16C3F is an ADC that specifically targets ENPP3. As shown in Table 1, a phase 2 study (NCT02639182) primarily focused on evaluating the efficacy and safety of AGS-16C3F in the treatment of mRCC patients [44]. The results indicated that AGS-16C3F did not achieve the expected goal. The AGS-16C3F group had an mPFS of 2.9 months, whereas the axitinib group had an mPFS of 5.7 months. Furthermore, there was no significant difference in the OS between the AGS-16C3F and axitinib groups. Based on these findings, the development of AGS-16C3F for mRCC was halted.

##### Anti-Trop-2 ADCs

Trop-2 is a transmembrane calcium signal transducer with potential as a valuable target for cancer therapy [45]. The IMMU-132-01 trial evaluated the safety and efficacy of SG, a novel ADC consisting of anti-Trop-2, in patients with a variety of advanced epithelial cancers (including RCC) who developed disease progression after treatment with at least one standard regimen [46]. The study demonstrated that SG was safe and effective in the treatment of solid tumors (treatment-related AEs [TRAEs] above grade 3 were rare except for treatment-related neutropenia), including RCC (Table 1). These findings highlighted the significance of Trop-2 as a broad target in the treatment of solid tumors.

There are some challenges in the application of ADCs in RCC, as was indicated by the results of the drug development and clinical trial. While there were many ADC targets identified in RCC, ADC-associated TRAEs can affect multiple organs throughout the body, and each drug has a different spectrum of AEs, depending on their different antibodies and cytotoxic drugs. For critical TRAEs linked to SG, such as neutropenia (57.8%) and diarrhea (56.2%) [46], hematological indicators, comorbidities, and organ function status should be evaluated in addition to routine physical examination (Eastern Cooperative Oncology Group [ECOG] performance status score). In the future, we look forward to further verifying the efficacy and safety of ADCs in RCC through larger clinical samples and more comprehensive clinical trials.

#### ICIs combined with ADCs in RCC therapeutics

The efficacy of immunotherapy alone is limited in first-line therapy, so the combination of immunotherapy and anti-vascular targeted therapy has become the focus of current research. With the development of tumor tissue-specific antigens of RCC, the combination of ADCs and ICIs targeting RCC is expected to become a new approach for the treatment of RCC, especially aRCC.

This combination therapy not only addresses the low reactivity of RCC to ICIs but also tackles the challenges of tumor-specific targeted therapy. At present, there is no clinical study on ADCs combined with ICIs for treating RCC. Nevertheless, given the characteristics of RCC and the mechanism of action of ADC/ICIs, a breakthrough in the treatment of aRCC can be expected.

### Monotherapy or combination therapy in UC

UC can develop in any part of the urinary tract. In 2020, more than 570,000 new cases of BC were diagnosed globally, resulting in approximately 200,000 deaths [47]. Currently, the treatment of BC primarily relies on surgery, radiotherapy, and chemotherapy, depending on the stage of the tumor [48]. In recent years, substantial progress has been made due to the development of technologies for personalized diagnosis and treatment, along with the advent of precision medicine [49]. Therefore, antibody-based targeted therapy, immunotherapy, and ADCs have emerged as key directions for UC drug treatment. EV, SG, and disitamab vedotin (RC48-ADC) have demonstrated promising anti-tumor activity and safety in the late-line treatment of locally advanced, unresectable, or metastatic UC (mUC).

#### Application of ICIs in UC therapeutics

UC, a tumor type known for immunogenicity, has been found to have several tumor-infiltrating immune cells and disease-associated mutations that enhance its immunogenicity [50]. The efficacy of ICIs is gradually being recognized due to the reporting of a large amount of clinical trial data. At present, these inhibitors are widely used in the treatment of BC, from second-line therapy to first-line therapy, and from adjuvant therapy to combination therapy, along with other regimens [51]. PD-1/PD-L1 inhibitors have shown great potential in the neoadjuvant therapy of BC. Studies have indicated that anti-PD-1 drugs not only improve the long-term clinical efficacy of second-line treatments but also improve patients’ quality of life [52]. The Food and Drug Administration (FDA) recently approved several PD-L1 inhibitors (atezolizumab, durvalumab, and avelumab) as well as PD-1 inhibitors (nivolumab and pembrolizumab) for the second-line treatment of locally advanced/mUC (la/mUC) that is first-line cisplatin intolerant [53]. Pembrolizumab and atezolizumab can also be used as a first-line therapy in patients who are cisplatin intolerant and have PD-L1 positive expression or are not candidates for any platinum-containing chemotherapy [54].

##### Anti-PD-1 inhibitors

ICIs are primarily used as a second-line treatment for la/mUC, but they have also shown efficacy against non-muscle–invasive BC (NMIBC). As shown in Table 2, anti-PD-1-related drugs, especially pembrolizumab, have been extensively studied in the field of UC. The KEYNOTE series of studies, which were recruited from 91 academic medical centers in 20 countries, laid the foundation for the use of pembrolizumab in the treatment of cisplatin-ineligible patients with advanced UC (aUC) who had not received chemotherapy. In the early stage, the KEYNOTE-045 study [55] demonstrated that pembrolizumab had a higher ORR (21.1% vs. 11.4%) and a longer mOS (10.3 months vs. 7.4 months) compared to the chemotherapy group, and fewer TRAEs of any grade were reported in the pembrolizumab group than in the chemotherapy group (60.9% vs. 90.2%). Subsequently, the KEYNOTE-052 study [56] found that pembrolizumab had anti-tumor activity and acceptable tolerance in la/mUC patients who did not tolerate cisplatin. In addition to muscle-invasive BC (MIBC), the KEYNOTE-057 study evaluated the efficacy of pembrolizumab in NMIBC that did not respond to Bacillus Calmette–Guerin (BCG). The results revealed that pembrolizumab monotherapy was tolerable and showed promising anti-tumor activity in patients with BCG-unresponsive NMIBC who refused radical cystectomy [57]. Given these results, pembrolizumab has become a new treatment option for patients who are cisplatin-ineligible or not suitable for chemotherapy, including the elderly, those with poor prognostic factors, and those with severe comorbidities.

**Table 2:** Clinical trials of ICIs, ADCs, and their combined application in UC.

Categories	Clinical trials	Phases	Patients	Drugs	Targets	Clinical outcomes	References
ICIs	KEYNOTE-045	3	52 la/mUC	Pemb	PD-1	Pemb vs. Chem: 2-year OS: 26.9% vs. 14.3%; PFS: 2.0 m vs. 4.9 m; ORR: 20.0% vs. 18.2%.	[55]
	KEYNOTE-052	2	370 mUC	Pemb	PD-1	Pemb appeared to be consistent regardless of age and/or status.	[56]
	KEYNOTE-057	2	334 NMIBC	Pemb	PD-1	Pemb was tolerable and showed promising anti-tumor activity.	[57]
	KEYNOTE-361	3	1010 aUC	Pemb	PD-1	PFS changed little; mPFS: 8.3 m vs. 7.1 m; mOS: 17.0 m vs. 14.3 m.	[58]
	POLARIS-03	2	151 mUC	Tori	PD-1	ORR: 26% with a DCR of 45%; mDR, PFS, and OS: 19.7 m, 2.3 m, and 14.4 m.	[59]
	NCT02527434	2	32 la/mUC	Tori	PD-1	ORR:18.8%, including CR: 6.3%, and PR: 12.5%.	[60]
	CheckMate 275	2	270 mUC	Nivo	PD-1	mOS:7.0 m.	[61]
	SWOGS1605	2	172 NMIBC	Atez	PD-L1	27% experienced a CR at 6.0 m; mDR: 17.0 m.	[63]
	IMvigor 211	3	127 la/mUC	Atez	PD-L1	mOS: 9.2 m vs. 7.7 m.	[64]
	IMvigor 210	2	123 la/mUC	Atez	PD-L1	ORR: 23%; CRR: 9%; mOS: 15.9 m.	[65]
	IMvigor130	3	1213 la/mUC	(A)Atez+C; (B)Atez; (C)C	PD-L1 + C	mPFS: 8.2 m(A), 6.3 m(C); mOS: 16.0 m(A), 13.4 m(C); mOS: 15.7 m(B).	[66]
	NCT02792192	2	24 NMIBC	Atez + BCG	PD-L1 + Immune targets	Atez + BCG was well-tolerated.	[67]
	NCT02516241	3	1032 la/mUC	Durv + Trem	PD-L1 + CTLA-4	This study did not meet either of its co-primary endpoints.	[70]
ADCs	RC48-C005	2	43 mUC	RC48-ADC	HER-2	ORR: 51.2%; mPFS: 6.9 m; OS: 13.9 m.	[75]
	KAMELEON	2	20 BC	T-DM1	HER-2	5 patients exhibited a PR; ORR: 38.5%.	[77]
	NCT01631552	2	515 cancers (including mUC)	SG	TROP-2	TRAEs: nausea (62.6%), diarrhea (56.2%), fatigue (48.3%), alopecia (40.4%), and neutropenia (57.8%).	[80]
	TROPHY-U-01	2	113 la/mUC	SG	TROP-2	ORR: 27%; MD of response: 7.2 m; mPFS: 5.4 m; OS: 10.9 m.	[81]
	NCT00462488	2	46 BC	OM	EpCAM	44% achieved a CR; Median time to recurrence: 274 and 408 days.	[85]
	EV301	3	608 la/mUC	EV	Nectin-4	EV vs. C: mOS: 12.88 m vs. 8.97 m; mPFS: 5.55 m vs. 3.71 m.	[86]
	EV201	2	219 la/mUC	EV	Nectin-4	ORR: 52%; 20% achieved a CR; 31% achieved a PR.	[88]
ICIs+ADCs	EV103 Cohort K	2	149 la/mUC	Pemb + EV	PD-1 + Nectin-4	ORR: 64.5% vs. 45.2%; mDOR of EV: 13.2 m.	[91]
	EV-302	3	45 la/mUC	Pemb + EV	PD-1 + Nectin-4	ORR: 73.3%; RR: 15.6%; mDOR: 25.6 m; mOS: 26.1 m.	[92]

However, not all KEYNOTE series studies have proven the satisfactory clinical effects of pembrolizumab. Data from the KEYNOTE-361 study [58] showed that adding pembrolizumab to first-line platinum chemotherapy did not significantly improve efficacy. Regarding the immunotherapy options for aUC patients after progression, there are several potential immune resistance mechanisms, including activation of other inhibitory checkpoint pathways and T-cell exhaustion [59, 60]. Later-line treatment strategies can consider targeting other coexisting driver genes or targeted treatments. Pembrolizumab can cause immune-related AEs. Because AEs may occur at any time during pembrolizumab treatment or after treatment is discontinued, patient monitoring should be continued.

Toripalimab is another anti-PD-1 drug. The POLARIS-03 trial evaluated the efficacy of toripalimab as a monotherapy in patients with mUC who did not respond to standard therapy. Notably, patients with PD-L1 positive expression and a higher tumor mutational burden (TMB) had a better ORR than those with PD-L1 negative expression and a lower TMB [61]. In a separate cohort (NCT02527434), 32 patients who previously did not respond to platinum-containing first-line chemotherapy were treated with toripalimab. The results showed an ORR of 18.8%, including two complete responses (CR) and four partial responses (PR) [62].

Nivolumab, another human monoclonal PD-1 antibody, was also investigated in the CheckMate 275 trial [63]. To our delight, the data showed that nivolumab monotherapy provided a meaningful clinical benefit regardless of PD-L1 expression, and had an acceptable safety profile in patients with previously treated metastatic or surgically unresectable UC.

It is worth noting that toripalimab has a high affinity for PD-1 (approximately 23 times that of pembrolizumab and 35 times that of nivolumab), which exerts a powerful and durable pathway blocking the disruption effect after binding [64]. In addition, toripalimab also undergoes stronger endocytosis than pembrolizumab and nivolumab, which can effectively reduce the expression of PD-1 on the cell membrane, so as to further enhance the PD-1 expression band, inhibit immunity, and promote “immune normalization” of tumors [65].

In the future, we look forward to clinical studies comparing the efficacy of these three PD-1 drugs in UC to help patients select the best drugs.

##### Anti-PD-L1 inhibitors

PD-L1, a ligand of PD-1, is known to play a role in suppressing the immune system and transmitting inhibitory signals [66]. The anti-PD-L1 drug atezolizumab has shown promising clinical effects in the treatment of UC. As shown in Table 2, the SWOGS1605 trial evaluated atezolizumab in patients with BCG-unresponsive NMIBC. Unfortunately, the results did not meet the predetermined efficacy threshold [67], and 26 patients (16%) experienced grade 3–5 TRAEs, including three treatment-related deaths. Therefore, when considering systemic immunotherapy for early-stage BC, modest efficacy needs to be balanced with significant rates of TRAEs and the risk of disease progression.

For patients with aUC who relapsed or progressed after platinum-based chemotherapy, atezolizumab significantly improved the prognosis. A phase 3 clinical trial (IMvigor211) [68] compared atezolizumab alone with chemotherapy (paclitaxel, docetaxel, or vinflunine). The mOS was higher in the atezolizumab group than in the chemotherapy group (8.6 months vs. 8.0 months). In addition, the latest data from the trial revealed that in the intention-to-treat population, atezolizumab had a 30-month OS of 18%, compared with 10% for chemotherapy. Similarly, the IMvigor 210 study [69] found that atezolizumab showed encouraging durable response rates, survival (2.7 months mPFS; 15.9 months mOS), and tolerability (TRAEs that occurred in 10% or more of patients were fatigue [30%], diarrhea [12%], and pruritus [11%]), supporting its therapeutic use in untreated mUC.

Although most TRAEs were grade 1 or 2, and immune-mediated events could be controlled with systemic corticosteroids alone, 8% of patients in this study stopped treatment due to AEs. Therefore, the response of different patients to atezolizumab deserves close attention, and active intervention or discontinuation of atezolizumab is necessary.

Overall, atezolizumab demonstrated a promising response, durability, survival, and a low incidence of clinically relevant toxicity, despite the presence of many comorbidities in this population.

##### Anti-PD-1 plus anti-PD-L1 inhibitors

Chemotherapy is the first-line treatment for MIBC. Whether ICIs combined with chemotherapy are better than chemotherapy alone has become a direction for researchers to explore. Based on the activity of atezolizumab in MIBC, the IMvigor130 study [70] tested atezolizumab + gemcitabine and cisplatin (GC) for MIBC (cT2-T4aN0M0). The researchers found that atezolizumab + GC was a promising regimen for MIBC and warranted further study. In addition, a phase 2 study (NCT02792192) investigated atezolizumab with or without BCG in patients with high-risk NMIBC. Preliminary results showed that atezolizumab in combination with BCG was well-tolerated, suggesting that this combination therapy may be beneficial in patients who do not respond to BCG [71].

The combination of two ICIs, especially PD-1/PD-L1 + CTLA-4, is also a focus area of clinical research. CTLA-4 is highly expressed in activated T cells and can inhibit their activities [72]. PD-1 and PD-L1 can play an immune escape role in TME, and PD-1/PD-L1 inhibitors can prevent T-cell immune escape and exhaustion of cancer cells [73]. The combination of the two drugs can improve the efficacy and produce a synergistic effect. The NCT02516241 study [74] evaluated the combination of durvalumab and tremelimumab in 1032 patients with untreated or unresectable la/mUC. The study revealed that in individuals with PD-L1 positive expression, the mOS in the combination group was similar to that in the durvalumab group (15.1 months vs. 14.4 months), but the incidence of AEs was higher.

From the above clinical trials, as a potential second-line treatment option for la/mUC, PD-1/PD-L1 combined immunotherapy provides better survival for patients who do not respond to initial therapy. However, combination therapy increases the risk of TRAEs and health care costs. Inappropriate combination therapy will expose patients to significantly higher toxicities. How to optimize the dosing regimen, including dosage, timing, and sequence, is another challenge in the development of combination therapy.

#### Application of ADCs in UC therapeutics

In recent years, ADCs have emerged as a promising treatment for UC, following immunotherapy and targeted therapy. Clinical studies of ADCs in UC have shown promising results. Whether used alone or in combination with traditional chemotherapy, ADCs bring new treatment options to patients. Several ADCs have been approved for the treatment of UC, mainly targeting human epidermal growth factor receptor-2 (HER-2), Trop-2, epithelial cell adhesion molecular (EpCAM), Nectin-4, and others.

##### Anti-HER-2 ADCs

HER-2 is a transmembrane glycoprotein belonging to the epithelial tyrosine kinase protein family, specifically the epidermal growth factor receptor (EGFR). It mediates signal transduction pathways such as the RAS/RAF/MAPK pathway and PI3K/AKT pathway [75]. Studies have demonstrated that HER-2 overexpression is associated with tumor cell proliferation, invasion, metastasis, and other factors. Approximately 10% to 20% of UC patients exhibit HER-2 positive expression [76], which is related to rapid disease progression, high chemotherapy resistance, easy recurrence after surgery, and shorter survival [77]. RC48-ADC is an anti-HER-2 drug developed by Rongchang Biological Company. It can overcome intratumoral heterogeneity and resist HER-2 overexpression caused by the growth of HER-2 negative clones [78]. In a phase 2 study of RC48-C005, 43 mUC patients who had previously failed at least one systemic chemotherapy received RC48-ADC monotherapy. The study reported an ORR of 51.2% and a disease control rate (DCR) of 90.7% during a median follow-up time of 20.3 months. The mPFS and OS were 6.9 months and 13.9 months, respectively [79].

Trastuzumab emtansine (T-DM1) is an anti-HER-2 drug that binds trastuzumab to cytotoxic drug emtansine (a tubulin inhibitor) through a thioether linker [80]. The KAMELEON study [81] demonstrated the safety and efficacy of T-DM1 in the treatment of HER-2-positive solid tumors (HER-2 in 30% or more of stained cells), including BC. However, due to the early termination of the project, the trial did not fully achieve its research objectives.

##### Anti-Trop-2 ADCs

Trop-2 overexpression is closely associated with increased disease aggressiveness in UC [82]. Therefore, trop-2 has become an emerging target in the field of precision medicine for UC. SG is the first approved ADC targeting Trop-2. Originally developed as a second-line treatment for triple-negative breast cancer, SG received FDA approval in April 2021, for the treatment of aUC (receiving platinum-containing chemotherapy) and la/mUC as a second-line treatment [83].

The NCT01631552 study [84] evaluated the efficacy of SG in six heavily pre-treated patients with metastatic platinum-resistant UC as early as 2016. Of these six patients, three had clinically significant responses (PFS 6.7 months to 8.2 months; OS more than 7.5 months to 11.4 months). This study highlights the promise of SG therapy for UC. The TROPHY-U-01 study enrolled 113 patients with la/mUC who had previously received platinum-based chemotherapy and PD-1/PD-L1 inhibitors. As expected, SG showed significant efficacy in the pre-treated mUC that had progressed in both previous platinum-based combination chemotherapy regimens and checkpoint inhibitors compared to controls (mPFS and OS; 5.4 months and 10.9 months, respectively.) [85].

##### Anti-EpCAM ADCs

EpCAM is a type I cell surface transmembrane glycoprotein that is overexpressed in a variety of tumor cells and is a target for cancer diagnosis and treatment [86]. It has multiple biological functions, including the acceleration of the cell cycle, promotion of cell proliferation, differentiation, migration, and immune escape [87]. OM is an ADC that consists of humanized single-chain antibody fragments targeting EpCAM and *pseudomonas aeruginosa* exotoxin A through genetic fusion [88].

An open-label, multi-center phase 2 clinical study (NCT00462488) [89] involved 46 patients with in-situ BC who had previously failed BCG treatment. These patients received different courses (6 or 12 weeks) of 30 mg OM intravesical infusion. The results showed that CR was similar between the two groups, but the median time to recurrence was longer after 12 weeks of treatment, suggesting that intensive therapy may be more beneficial.

Considered together, OM demonstrated preliminary anti-tumor activity and was well-tolerated in NMIBC patients who had previously received BCG. However, further clinical data are needed to support the specific clinical efficacy and drug safety of OM.

##### Anti-Nectin-4 ADCs

Nectin-4, a type I membrane protein, is found to be overexpressed in various malignant tumors including UC and breast cancer. Its mechanism involves the activation of the PI3K/AKT pathway to promote tumor cell proliferation, differentiation, invasion, and metastasis [90]. EV is an ADC targeting Nectin-4, consisting of a fully human monoclonal antibody against Nectin-4 and monomethyl auristatin E (MMAE), which induces cell cycle arrest and apoptosis, leading to tumor cell death [91].

The EV-301 trial explored la/mUC patients who had previously received platinum-containing chemotherapy and developed disease progression during or after treatment with PD-1 or PD-L1 inhibitors. The results confirmed that EV significantly prolonged survival compared to standard treatment (mOS: 12.88 months vs. 8.97 months, mPFS: 5.55 months vs. 3.71 months) [90]. Similarly, the EV-201 study showed that EV therapy was tolerable and confirmed the efficacy in 52% of cisplatin-ineligible la/mUC patients, but 55% of patients had grade 3 or worse TRAEs [92]. Therefore, EV can be considered as a new therapy for patients who have failed to respond to previous chemotherapy or ICI treatment.

Although EV has shown significant efficacy, the high incidence of peripheral neurotoxicity and severe cutaneous toxicity after intravenous administration has been linked to its use in first-line patients [93]. However, the local exposure characteristics of intravesical therapy significantly reduce the systemic exposure dose, which may open up new possibilities for EV in the treatment of NMIBC [94].

With the emergency of personalized diagnosis and treatment technology and the concept of precision medicine, ADCs have emerged in the treatment of UC, providing a very promising treatment strategy for patients, especially in the treatment of patients who have failed immunotherapy in the past, showing good results and safety. However, the complexity of ADCs also makes their applications face many challenges, such as the regulation of antigen recognition by antibodies may create new mechanisms, leading to drug resistance in some patients [95]. In addition, the optimization of various components of ADCs remains to be further explored, including how to use fully humanized monoclonal antibodies to reduce immunogenicity, how to utilize cytotoxic drugs with different mechanisms of action, and how to optimize linker design and linking methods to reduce off-target toxicity [96, 97].

#### ICIs combined with ADCs in UC therapeutics

Extensive studies of ADCs in the treatment of UC have indicated that its primary efficacy is in combination therapy rather than monotherapy. The ideal drugs to combine with ADCs are those that have complementary or synergistic effects on tumor cells or their microenvironment, without causing excessive overlapping toxicities. Like most cytotoxic drugs, the duration of a positive response or clinical benefit of ADCs as standalone therapy is limited due to the development of resistance mechanisms. Therefore, the combination of ADCs with other anticancer drugs has become a crucial focus of ADCs development. Clinical trials of combined immunotherapy and ADCs have recently emerged, and there is growing evidence that ADCs can enhance the efficacy of immunotherapy drugs.

EV combined with pembrolizumab is the most studied ICIs-ADCs combination model. The EV-103 Cohort K study tested 149 cisplatin-ineligible patients with previously untreated la/mUC who were randomly assigned 1:1 to receive EV monotherapy or a combination with pembrolizumab. In fact, EV + pembrolizumab showed a high confirmed ORR (64.5% vs. 45.2%) and durable response in la/mUC patients. AEs of double drugs were controllable, and no new safety signals were observed [98]. Another phase 3 trial (EV-302) also investigated this combination therapy in 45 cisplatin-ineligible patients and revealed that EV combined with pembrolizumab had an ORR of 73.3% and a CR of 15.6%. The safety of combination therapy was similar to that of monotherapy, although the ORR was higher [99]. Therefore, the combination of EV and ICIs has great prospects for the future treatment of aUC.

#### Ongoing clinical trials in UC

In addition to the results of the above-mentioned clinical trials mentioned above, there are many exciting and important studies underway. These studies include immunotherapy and its combination therapy, ADCs, and immunotherapy combined with ADCs. As shown in Supplementary Table S1, the MK-3475-992 trial is ongoing. To further clarify the role of pembrolizumab combined with chemoradiotherapy vs. chemoradiotherapy in MIBC, substantial evidence supports the advantages of additional front-line treatments.

There are more ongoing trials on ADCs in UC not yet published their results. In Supplementary Table S1, the RC48-C009 and RC48-C014 studies are also further exploring the efficacy of RC48-ADC (anti-HER-2) in UC. The NCT04073602 study is being studied for RC48-ADC in HER-2 negative la/mUC patients. Furthermore, in addition to RC48-ADC monotherapy, another trial, NCT05016973, is exploring RC48-ADC in combination with triplizumab for MIBC. As anti-Trop-2, IMMU-132-13, and SURE-01 are several SG-related phase 2 and 3 clinical studies in progress. In addition, the NCT02449239 study further established the safety and efficacy of OM (Anti-EpCAM) in the treatment of NMIBC previously treated with BCG. Besides OM, another phase 2 clinical trial (NCT04859751) of VB4-845 (another anti-EpCAM ADC) is also ongoing, and the results have not yet been released. These studies will further supplement the shortcomings and limitations of previous ADCs in UC applications. In the future, the results of these studies will provide more options for UC patients.

It is clear from these clinical studies that ADCs show improved results when combined with ICIs. This synergy between the two drugs enhances their ability to kill tumors. In addition to the clinical trials with clear efficacy, there are many ongoing ICIs plus ADCs-related phase 2/3 clinical trials, such as pembrolizumab + EV (NCT05239624, NCT03924895, NCT04700124, and NCT03606174), durvalumab/tremelimumab + EV (NCT04960709), and atezolizumab + EV (NCT03869190), etc. (Supplementary Table S1). The publication of these studies in the future will offer additional treatment options for the combined use of ICIs plus ADCs. In addition to monotherapy and dual-drug combination, three-drug combination clinical trials are also being explored. The NCT04863885 is a phase 2 clinical trial studying the combination of nivolumab + ipilimumab + SG in metastatic cisplatin-ineligible BC. While multi-drug combination therapy may improve clinical efficacy, it is important to recognize that the toxic side effects of these drugs may also be exacerbated. This aspect deserves careful consideration. Therefore, it is of great clinical significance to find new, safe, and effective therapeutic methods to improve the prognosis of patients.

### Monotherapy or combination therapy in PCa

PCa is the second most common cancer in the world and the fifth leading cause of cancer death in men, with approximately 1.4 million cases and 375,000 deaths worldwide in 2020 [100]. Common treatments for PCa include surgery, endocrine therapy, chemotherapy, and radiotherapy. Androgen deprivation therapy (ADT) serves as the foundation and standard of all treatments [101]. However, a major challenge is that most patients develop hormone resistance soon after receiving endocrine therapy [102]. This resistance not only complicates PCa treatment but also increases the risk of death [103]. In addition, due to the dense capsule structure of prostate tissue, many drugs have difficulty reaching the interior of the prostate to effectively target tumors [104]. This poses a significant challenge for the drug treatment of PCa. As a result, a variety of new targeted drugs specifically designed for PCa are constantly being developed.

#### Application of ICIs in PCa therapeutics

PCa exhibits the following immune characteristics: (1) T-cell infiltration is decreased while PD-1 expression is increased. (2) Advanced PCa (aPCa) presents a higher proportion of Tregs. (3) Tumor-associated macrophages (TAM) are mainly composed of M2 TAM. ICIs have been used in the treatment of aPCa, but have not shown significant efficacy, which indicates the need to further study the mechanism of ICIs in PCa [105].

##### Anti-PD-1/PD-L1 inhibitors

In the treatment of PD-1/PD-L1 positive aPCa patients, PD-1/PD-L1 inhibitors have shown promising results [106]. One of these inhibitors is pembrolizumab, a humanized anti-PD-1 monoclonal antibody. The KEYNOTE-199 study consisted of three cohorts of patients with metastatic castration-resistant PCa (mCRPC) who were treated with docetaxel and one or more targeted endocrine therapies. Enrolled in cohorts 1 and 2 were patients with RECIST-measurable PD-L1-positive and PD-L1-negative disease, respectively. Enrolled in cohort 3 were patients with bone-predominant disease, regardless of PD-L1 expression. The study found that in mCRPC patients treated with chemotherapy and ADT, pembrolizumab monotherapy showed significant anti-tumor activity and a favorable safety profile (ORR of 5% in cohort 1 and 3% in cohort 2). CR was 10% in cohort 1, 9% in cohort 2, and 22% in cohort 3. MOS was 9.5 months in cohort 1, 7.9 months in cohort 2, and 14.1 months in cohort 3 [107].

Ipilimumab is an anti-CTLA-4 inhibitor that expands the range of available tumor immunotherapy approaches, in contrast to the widespread use of single-drug PD-1/PD-L1 inhibitors in the field of tumor therapy. To determine the efficacy of ipilimumab in PCa, the investigators conducted several phase 2/3 clinical trials (Table 3). The CA184-043 study [108] is a multi-center, randomized, double-blind, phase 3 trial in which at least one patient with CRPC bone metastasis progressed after docetaxel treatment. It was found that although there was no significant difference in OS between the ipilimumab group and the placebo group (11.2 months vs. 10.0 months), there were signs of drug activity that warrant further investigation.

**Table 3:** Clinical trials of ICIs, ADCs, and their combined application in PCa.

Categories	Clinical trials	Phases	Patients	Drugs	Targets	Clinical outcomes	References
ICIs	KEYNOTE-199	2	388 mCRPC	Pemb	PD-1	ORR: 5% vs. 3%; mOS: 9.5 m vs. 7.9 m.	[100]
	CA184-043	3	799 mCRPC	Ipil	CTLA-4	mOS: 11.2 m vs. 10.0 m.	[101]
	CA184-095	3	837 mCRPC	Ipil	CTLA-4	mOS: 28.7 m vs. 29.7 m; mPFS: 5.6 m vs. 3.8 m.	[102]
	KEYNOTE-365 Cohort B	2	104 mCRPC	Pemb + C	PD-1	mPFS: 8.5 m; OS: 20.2 m; 78% DR.	[103]
	NCT02312557	2	58 mCRPC	Pemb + Enza	PD-1 + ADT	18% had a PSA decline of ≥50%; 25% achieved an OR.	[104]
	KEYLYNK-010	3	529 mCRPC	Pemb + Olap	PD-1 + PARP	mOS: 15.8 m vs. 14.6 m; meTFST: 7.2 m vs. 5.7 m.	[106]
	KEYNOTE-365 Cohort A	2	102 mCRPC	Pemb + Olap	PD-1 + PARP	cORR: 8.5%; mrPFS: 4.5 m; mOS: 14.0 m.	[107]
	CheckMate 650	2	351 mCRPC	Nivo + Ipil	PD-1 + CTLA-4	ORR: 25% vs. 10%; mOS: 19.0 m vs. 15.2 m.	[108]
	NCT02499835	2	25 mCRPC	Pemb + MVI-816	PD-1+T-cell	Radiographic PFS: 47.2%; mOS: 22.9 m.	[109]
ADCs	M59102-042	2	29 mCRPC	MLN2704	PSMA	MLN2704 can be administered safely.	[112]
	NCT00070837	2	62 mCRPC	MLN2704	PSMA	8% PSA declined ≥50%; 8% PSA stabilization ≥90 days.	[113]
	NCT01695044	2	119 PCa	PSMA-ADC	PSMA	14% PSA declined ≥50%; 78% CTC declined ≥50%.	[114]
	NCT01812746	2	42 mCRPC	BIND-014	PSMA	Median radiographic PFS: 9.9 m.	[115]
	NCT02923180	2	32 PCa	NE	B7-H3	Feasible and generally safe, has potential clinical activity.	[118]
	NCT01774071	2	19 mCRPC	89Zr-DFO-MSTP2109A	STEAP1	No significant toxicity occurred.	[121]
	Innova TV 201	2	27 CRPC	TV	TF	TV has a safety profile with preliminary anti-tumor activity.	[123]
	NCT02709889	1/2	135 NEPC	Rova-T	CD19	Rova-T can improve drug delivery, reduce toxicity, and increase treatment duration.	[124]
ICIs+ADCs	NCT03406858	2	14 mCRPC	Pemb + HER2-BATs	PD-1 + HER-2	PFS: 6 m in 5/14 patients; mPFS: 5.0 m; median survival: 31.6 m.	[126]
	KEYNOTE-046	1/2	50 mCRPC	Pemb + ADXS31142	PD-1 + PSA	mPFS: 2.2 m vs. 5.4 m; mOS: 7.8 m vs. 33.7 m.	[127]

In 2017, another study (CA184-095) was conducted on patients with mCRPC who were asymptomatic or mildly symptomatic and did not receive chemotherapy [109]. The results showed no statistically significant difference in OS, with a PFS of 5.6 months in the ipilimumab group and 3.8 months in the placebo group. Besides, the most common grade 3–4 AEs were immune-related, occurring in 101 patients (26%) in the ipilimumab group and 11 patients (3%) in the placebo group in this trial. Therefore, the efficacy and safety of ipilimumab in PCa need to be further evaluated through clinical trials with larger samples.

##### Anti-PD-1 plus PD-L1 inhibitors

For patients with aPCa or CRPC, ICIs in combination with other treatments have shown enhanced clinical benefits. The KEYNOTE-365 Cohort B study evaluated the efficacy and safety of pembrolizumab + chemotherapy in patients with mCRPC [110]. Remarkably, the combination of pembrolizumab with docetaxel showed antitumor activity, and safety was consistent with that of individual agents. Additionally, pembrolizumab in combination with enzalutamide was active in mCRPC after prior enzalutamide treatment, and the response was deep and long-lasting, without requiring tumor PD-L1 expression or DNA-repair defects (NCT02312557) [111].

Olaparib is the first approved PARP inhibitor that effectively targets the DNA damage repair response pathway and employs the concept of “synthetic lethality” to selectively eliminate cancer cells while minimizing harm to healthy cells. It is specifically approved for mCRPC patients with homologous recombination repair gene mutations who have been treated with androgen receptor antagonists enzalutamide or abiraterone [112]. The KEYLYNK-010 trial [113] showed that pembrolizumab + olaparib did not significantly improve PFS (4.4 months vs. 4.2 months) and OS (15.8 months vs. 14.6 months) in mCRPC patients without selected biomarkers, compared with next-generation hormonal agents. However, the incidence of grade 3 or more TRAEs was 34.6% and 9.0%, respectively. As a result, the study was stopped due to its ineffectiveness.

In contrast, the KEYNOTE-365 Cohort A [114] showed a proven safety profile of pembrolizumab in combination with olaparib and demonstrated antitumor activity in patients with mCRPC who had previously received chemotherapy. The confirmed ORR of patients with measurable disease was 8.5%; the median rPFS was 4.5 months; and the mOS was 14 months. Although limitations of this study include the single-arm design, it laid the foundation for pembrolizumab in combination with olaparib for mCRPC. Furthermore, the “PD-1 + CTLA-4” model has also been applied in the treatment of mCRPC. The CheckMate 650 study observed the effect of nivolumab combined with ipilimumab in mCRPC, and found that the combined therapy showed anti-tumor activity (ORR of 25% and 10% in cohorts 1 and 2, mOS of 19.0 and 15.2 months, respectively), preliminarily demonstrating a potential biomarker response [115].

These early data support further evaluation of ICI-based combinations in mCRPC patients, but questions remain about the optimal dose/regimen. However, the most common grade 3–4 TRAEs were diarrhea, pneumonitis, and increased lipase, which should not be taken lightly. Continued analyses of nivolumab plus ipilimumab, focusing on dose optimization and further evaluation of biomarkers, as well as other ongoing trials, will provide relevant data for the development of effective immunotherapy strategies for the treatment of PCa patients.

In recent years, the combination of ICIs and PCa vaccines has become a research hotspot. A phase 2 trial (NCT02499835) investigated T-cell activation of pembrolizumab and MVI-816, a DNA vaccine encoding prostatic acid phosphatase (PAP), in mCRPC. The combined therapy was found to be safe, with an estimated overall radiographic PFS rate of 47.2% at 6 months and mOS of 22.9 months [116] The results also showed that immune-related AEs (irAEs) were observed in 42% of patients, which were significantly associated with prolonged treatment. It was also observed that this combination can enhance the presence of tumor-specific T cells, leading to a favorable 6-month DCR. Relevant studies indicated that activation by inoculation of T cells plays a critical role in the mechanism of action of this combination [21]. However, further randomized clinical trials are needed to validate these findings.

As a “cold” tumor, the PD-1/PD-L1 pathway is not the only speed-limiting factor of anti-tumor immunity for PCa, and blocking the PD-1/PD-L1 axis alone is not enough to stimulate an effective anti-tumor-immune response. Therefore, some combination therapies, including PD-1/PD-L1 plus ADT, chemotherapy, radiotherapy, or other ICIs, have better anti-tumor efficacy and higher response rates.

#### Application of ADCs in PCa therapeutics

In recent years, with the development of protein-related technologies, several tumor-specific antigens related to PCa have been identified. These antigens include prostate-specific antigen (PSA), PAP, and prostate-specific membrane antigen (PSMA), et al. These antigens may be ideal targets for targeted therapy of PCa. Currently, important targets for PCa include PSMA, B7-H3, prostate six-transmembrane epithelial antigen 1 (STEAP1), tissue factor (TF), CD19, et al.

##### Anti-PSMA ADCs

PSMA is a type II transmembrane glycoprotein that is predominantly expressed in the cytoplasm and epithelial apex around the prostate ducts. During the occurrence of PCa, PSMA metastasizes to the luminal surface of the ducts, showing high specificity for PCa [117]. A study revealed that PSMA levels in PCa were 1,000 times higher than in benign prostate tissue [118]. Several ADCs targeting PSMA have been developed, including MLN2704, PSMA-ADC, and BIND014.

The M59102-042 study [119] confirmed the safety and reliability of MLN2704 in mCRPC patients. Of these, 13% had grade 3 TRAEs, and no grade 4 TRAEs was observed. However, the limitation of this study is that only the safety of MLN2704 was assessed, and the optimal dosage and frequency of administration were unclear. Consequently, NCT00070837, a follow-up study based on the results of M59102-042, is a phase 1/2 clinical trial examining the dosage, pharmacokinetics, immunogenicity, and tumor response of MLN2704 in 62 patients with mCRPC. The results revealed that MLN2704 had limited activity in mCRPC, with only 2 out of 35 patients experiencing PSA reductions greater than 50% over the 3- and 6-week regimens. This may be due to the instability and rapid unbundling of disulfide junctions leading to neurotoxicity and a narrow therapeutic window [120].

To evaluate the efficacy of PSMA-ADC in mCRPC whose disease progressed after abiraterone/enzalutamide treatment, Petrylak et al. [121] conducted a study (NCT01695044) involving 119 participants. The results showed that PSMA-ADC demonstrated some activity in PSA reduction and circulating tumor cell conversion. Among them, PSA decreased by more than 50% in 14% of all treated and 21% of chemotherapy-native subjects, and CTC decreased by more than 50% in 78% of all treated and 89% of chemotherapy-native subjects. The most common serious AEs were dehydration, hyponatremia, febrile neutropenia, and constipation.

BIND-014, a drug containing docetaxel and targeting PSMA, was studied in 42 chemotherapy-naive mCRPC patients whose disease progressed after treatment with abiraterone and/or enzalutamide (NCT01812746). The results demonstrated that BIND-014 treatment was effective in previously untreated mCRPC patients, with 30% having a PSA response and 32% having a measurable disease response [122].

However, toxicity (31.3%) was the most common reason for the discontinuation of these anti-PSMA ADC therapies. Two deaths from sepsis and neutropenia were reported to be directly related to the investigatory drug. These AEs are all dose-dependent, and the optimal effective concentration and toxic dose require more detailed studies to ensure their safety.

##### Anti-B7-H3 ADCs

B7-H3 is considered a more valuable target in PCa, particularly in the context of CRPC and castration-sensitive PCa. Biopsies of these types of PCa often show expression of both membranous B7-H3 (134/141, 95.0%) and cytoplasmic B7-H3 (137/141, 97.2%), indicating that targeting these two forms of B7-H3 could significantly improve the efficacy of PCa targeted therapy [123]. Notably, Shi et al. [124] confirmed the effectiveness of B7-H3 as a therapeutic target in PCa and proposed the use of PTEN/TP53 as a biomarker to guide the use of B7-H3 targeted drugs, thus providing a potential regimen for PCa.

Neoadjuvant enoblituzumab (NE), a humanized ADC targeting B7-H3, was evaluated in a phase 2 clinical study (NCT02923180). The study enrolled 32 patients with operable medium-high risk localized PCa to evaluate the safety, anti-tumor activity, and immunogenicity of NE given before prostatectomy. The co-primary endpoint for the rate of PSA_0_ (undetectable PSA level) at 1 year after postprostatectomy was 66%, and the primary safety endpoint was met with no notable unexpected surgical or medical complications, nor surgical delays [125]. The current study validates that B7-H3 is a reasonable target for PCa treatment, and larger studies are planned. Based on these findings, the use of B7-H3 targeted immunotherapy in PCa is feasible and generally safe, with preliminary data showing potential clinical activity.

##### Anti-STEAP1 ADCs

STEAP1 is predominantly expressed in PCa cells with low or no expression in normal tissues, making it a potential cell surface target for imaging and therapeutic intervention [126]. Immunohistochemistry confirmed its high expression in prostate epithelial cells, especially at cell–cell junctions [127]. The research and development of STEAP1 targets is still in its early stages.

89Zr-DFO-MSTP2109A is a compound obtained by conjugating microtubule inhibitor MMAE with the STEAP1 antibody. Preclinical studies (NCT01774071) showed that 89Zr-DFO-MSTP2109A was well-tolerated, without significant toxicity and could be localized in bone and soft tissue of mCRPC patients [128]. Given its highly standardized uptake value localization in tumors and the presence of numerous lesions, this agent warrants further exploration and has the potential to be used as a companion diagnosis in patients receiving STEAP1-directed therapy in the future.

##### Anti-TF ADCs

TF is a transmembrane glycoprotein, which is the main initiator of the exogenous coagulation pathway. It is also involved in cell signaling processes associated with adverse clinical outcomes such as tumor growth, angiogenesis, and metastasis [129].

Tisotumab vedotin (TV) is the first ADC that targets TF. Innova TV 201 is a phase 1/2 open-label, dose-escalation, and dose-expansion study conducted at 21 centers in the United States and Europe to evaluate the safety and efficacy of TV in patients with solid tumors, including PCa. Unfortunately, significant dose-limiting toxicities were observed in the clinical trials, including grade 3 TRAEs, type 2 diabetes mellitus, mucositis, and neutropenic fever [130]. Therefore, it is crucial to further monitor the specific efficacy and AEs of this drug in different tumors in the subsequent stage.

##### Anti-CD19 ADCs

Loncastuximab tesirine (Rova-T), an ADC targeting CD19, has been found to be effective against solid tumors, including neuroendocrine PCa, in the NCT02709889 trial [131]. Although the primary endpoint was safety, grade 3/4 AEs, including anemia (17%), thrombocytopenia (15%), and elevated aspartate aminotransferase (8%), warrant further testing using future large-sample studies.

#### ICIs combined with ADCs in PCa therapeutics

For PCa with low PD-1 expression, most patients do not respond to immunotherapy [132]. Therefore, it has become crucial to explore novel therapeutic modalities, such as the combination of ICIs and ADCs, which have the potential to address the “cold” tumor characteristics of PCa.

The NCT03406858 trial was conducted to evaluate the safety and efficacy of pembrolizumab in combination with HER-2 bi-specific antibody (HER2Bi)-armed activated T cells (HER2 BAT). As a result, 5 of 14 patients (38.5%) achieved a PFS of 6 months, mPFS of 5 months, and mOS of 31.6 months. Furthermore, AEs were grade 1–2 infusion reactions with fever, chills, headaches, nausea and/or myalgias [133].

In addition, the KEYNOTE-046 study evaluated ADXS31-142, an attenuated listeria monocytogenes-based immunotherapy targeting PSA, as a monotherapy and in combination with pembrolizumab for mCRPC [134]. As expected, ADXS31-142 in combination with pembrolizumab was safe in mCRPC patients and deserved a further evaluation for OS improvement (33.7 months vs. 7.8 months), especially in patients with visceral metastases. Thankfully, no additive toxicity was observed with the combination treatment.

In the future, it is critical to gain a deeper understanding of the IME of PCa, and this understanding will allow us to explore the various factors that influence immunotherapy. We should also focus on optimizing patient selection strategies to overcome immunosuppression and immune escape through innovative combination and sequence therapy to improve the effectiveness of immunotherapy in mCRPC. In addition, it is important to continue research and development in ADCs. These drugs can not only deliver targeted small-molecule cytotoxic drugs to tumors but also have the potential to regulate immunity and facilitate the transformation of “cold” tumors into “hot” tumors. This transformation will be beneficial in combination with ICIs to improve clinical outcomes.

#### Ongoing clinical trials in PCa

Currently, the medical and scientific community is engaged in a variety of ongoing clinical trials that are specifically focused on the application of ADCs in the treatment of PCa. As in Supplementary Table S2, several ongoing trials evaluating B7-H3-targeted ADCs for PCa (NCT04145622, NCT03729596, and NCT05551117) were designed to rigorously test the efficacy and safety of ADCs. In the future, the results and data emerging from these trials will not only be crucial for the immediate evaluation of ADC’s potential role in PCa treatment but are also expected to provide invaluable insights that will shape the future landscape of cancer therapeutics. The findings will inform researchers and clinicians about the most effective ways to use ADCs, the specific patient populations that are most likely to benefit, and the optimal combination therapies that can enhance their efficacy. Additionally, the ongoing trials are also likely to shed light on the long-term side effects of ADCs, the economic implications of incorporating these therapies into standard care, and the ethical considerations surrounding their use. In summary, the ongoing trials of ADCs in PCa represent a significant frontier in cancer research. They hold the promise of not only improving the lives of those affected by PCa but also advancing our collective understanding of cancer.

### Monotherapy or combination therapy in TGCT

TGCT is the most common solid malignant tumor in young men aged 20 to 34 years [135]. In TGCT, testicular seminoma is the most prevalent, accounting for nearly half of all cases [136]. Cisplatin-based chemotherapy has been proven to successfully treat approximately 90% of TGCT [137]. However, this therapy is associated with an increased risk of secondary cancer and cardiovascular disease [138]. Recent studies have shown that testicular seminoma is rich in immune cells, suggesting that immunotherapy may be a potential alternative treatment option [139]. Furthermore, the targeted therapy for TGCT also showed promising outcomes [140].

#### Application of ICIs in TGCT therapeutics

A notable feature of TGCT is the large number of immune cells in the TME, including lymphocytes, macrophages, mast cells, natural killer cells, and dendritic cells [141]. This abundance of immune cells has led to the consideration of ICIs as the preferred potential treatment for this type of tumor. Preclinical studies have shown that PD-L1 is expressed in 73% of seminomas and 64% of non-seminomas [142]. These studies also suggested that tumor-infiltrating lymphocytes expressing PD-L1 in seminomas may have a prognostic effect. Moreover, PD-L1 expression on TGCT is considered to have prognostic value, indicating that patients with high PD-L1 expression are more likely to exhibit poorer clinical features and survival outcomes [143].

##### Anti-PD-1 inhibitors

However, despite the encouraging results of laboratory studies, the application of ICIs in TGCT in some clinical trials has not produced favorable results. As shown in Table 4, the GU14-206 study [144] was the first immunotherapy study in TGCT, a single-arm phase 2 trial that investigated 12 patients with relapsed TGCT with no curable options. As a result, six patients had late recurrence (>2 years), and no irAEs were reported. These findings suggested that pembrolizumab was well-tolerated, but did not appear to have clinically meaningful monotherapy activity in refractory TGCT.

**Table 4:** Clinical trials of ICIs, ADCs, and their combined application in TGCT.

Categories	Clinical trials	Phases	Patients	Drugs	Targets	Clinical outcomes	References
ICIs	GU14-206	2	12 TGCT	Pemb	PD-1	mAFP: 615 μg/L; hCG: 4.6 mIU/ml; late relapse (>2 years).	[137]
	NCT03403777	2	8 TGCT	Avel	PD-L1	12-week PFS: 0%; mPFS: 0.9 m; mOS: 2.7 m.	[138]
	UMIN000028249	2	17 TGCT	Nivo	PD-L1	Nivo was well-tolerated, with only two Grade 3 AV.	[140]
ADCs	NCT01461538	2	40 TGCT	BV	CD30	2/7 patients achieved an OR, including one durable CR and one PR at a single time point.	[143]
	NCT02689219	2	18 TGCT	BV	CD30	mAFP: 4.9 μg/L; hCG: 282.5 mIU/ml; late relapse (>2 years).	[145]

##### Anti-PD-L1 inhibitors

Another phase 2 clinical trial (NCT03403777) evaluated the efficacy and safety of avelumab in TGCT. The results showed that avelumab was well-tolerated, and no serious AEs were observed. Unfortunately, all patients experienced disease progression, and neither OS nor PFS met their primary endpoints (2.4 months for mPFS and 10.6 months for mOS). These results indicated that avelumab was ineffective in unselected multiple relapsed/refractory TGCT [145]. Besides, Zschabitz et al. [146] analyzed the responses to ICIs in patients with refractory TGCT from 2015 to 2017. Among them, seven patients were treated with nivolumab or pembrolizumab, and four patients experienced tumor progression shortly after receiving a single dose and subsequently died from the disease. However, given the limited selection of assessment criteria in this study, larger-scale prospective clinical trials are needed to validate this finding.

Nivolumab is also used to treat TGCT. A phase 2 trial (UMIN000028249) [147] evaluated 17 patients with primary refractory TGCT after second-line or subsequent chemotherapy. One patient showed PR, and 3 patients demonstrated disease stabilization. The responses in one PR patient and one stable patient were durable, with a median duration of 90 and 68 weeks, respectively.

In conclusion, ICIs are not an ideal treatment for TGCT, and larger prospective clinical trials are needed. The development of personalized treatment based on genetic testing for individual patients may be a new therapeutic approach for TGCT.

#### Application of ADCs in TGCT therapeutics

The suboptimal response of ICIs in TGCT and the non-negligible impact of chemotherapy on TGCT encourage the study of ADCs in TGCT. CD30, a transmembrane glycoprotein of the TNF receptor family, is highly expressed in some non-germ cell reproductive tumors and in 93%–98% of testicular embryonic carcinomas, with specific effects on tumor cell proliferation [148]. Brentuximab vedotin (BV) is an ADC targeting CD30, consisting of a CD30 antibody conjugated to the MMAE. After BV is internalized into the lysosomes of tumor cells, MMAE binds to microtubules, leading to disruption of the intracellular microtubule network, followed by apoptosis of CD30-expressing cells [149]. BV has shown strong clinical activity in CD30-expressing tumors, such as classical Hodgkin’s lymphoma, anaplastic large-cell lymphoma, mycosis fungoides, and peripheral T-cell lymphoma [150].

Based on the above promising clinical results, a clinical trial (NCT01461538) [151] evaluated the clinical activity of BV in CD30-expressing non-lymphoma malignancies, including TGCT. The results discovered that two of seven patients achieved an objective response, including one durable CR and one PR at a single time point, and that BV treatment was generally well-tolerated. Therefore, BV may be a treatment option for this particularly aggressive disease. Another phase 2 clinical trial (NCT02689219) evaluating BV for relapsed/refractory TGCT indicated that six patients achieved radiographically stable disease (range 9 to 14.9 weeks); five patients had a transient reduction of greater than 50% in AFP or hCG at baseline; 10 patients showed an optimal response to disease progression, and two patients could not assess the response, meaning that no CR was observed [152]. This suggests that BV does not appear to have clinically significant monotherapy activity in relapsed/refractory TGCT.

#### ICIs combined with ADCs in TGCT therapeutics

Given the limited role of ICIs in TGCT, the researchers attempted to explore the potential of ICIs combined with ADCs to treat TGCT. The NCT06041503 study (Supplementary Table S3) is a phase 2 clinical trial evaluating the efficacy of BV with or without pembrolizumab in TGCT. A total of 68 participants were enrolled in this study, which is still in the research phase. The primary outcome measure was ORR, and the secondary outcomes were PFS and PR. With the continuous development of new targets for TGCT, we expect more combination therapy regimens to enter clinical trials in the future.

### Monotherapy or combination therapy in PeCa

PeCa is a rare malignant tumor, with the main histological type being penile squamous cell carcinoma (PSCC). Approximately 42% to 48% of PSCC cases is associated with HPV infection [153]. In the United States, there are approximately 0.5 to 2.1 cases per 100,000 men, while in regions such as Asia, Africa, and South America, the incidence is about 1% to 2% [154]. Early-stage patients have a better prognosis after surgery, while patients with pelvic and distant metastases have a 5-year survival rate of less than 10%, requiring combination therapy [155].

#### Application of ICIs in PeCa therapeutics

The study of the IME provides valuable insights into the immunological mechanisms of PeCa progression and metastasis, which can guide the development of immunotherapy strategies [156]. Several studies have reported high expression of PD-L1 in PSCC, supporting the potential use of ICIs in PSCC. Recent studies have demonstrated that PD-L1 expression is elevated in 48% to 62% of penile tumors, with a higher prevalence in HPV-negative cases [157]. Published data on the use of ICIs in PeCa are very limited and are mainly small case studies in patients with metastatic disease who have relapsed after the first-line chemotherapy.

Currently, there are no completed clinical trial data on ICIs for PSCC, and several ongoing clinical trials are investigating the role of ICIs in different stages of PSCC. The NCT04224740 and NCT02721732 studies are investigating the efficacy of pembrolizumab in PeCa (Supplementary Table S4). In addition to the anti-PD-1 drug, a phase 2 clinical trial (NCT03391479) is evaluating the impact of an anti-PD-L1 drug, avelumab, in patients with locally advanced or metastatic PeCa. These patients were ineligible for platinum-based chemotherapy or disease progression after platinum-based chemotherapy, and the primary study endpoint was assessed by ORR. Another anti-PD-L1 drug, atezolizumab, is currently in a phase 2 clinical trial (NCT03686332). These results will provide valuable insights into the effectiveness of ICIs in treating PeCa.

Overall, there are limited clinical studies on ICI treatment for PSCC at present, and most studies are still in progress. We eagerly look forward to the publication of these results to further inform our PSCC treatment.

#### Application of ADCs in PeCa therapeutics

Although there are currently limited large-scale clinical studies using ADCs in the treatment of PeCa, based on the understanding of the pathogenesis and signaling pathways, ADCs may become a novel therapeutic approach for PeCa in the future. First, with advances in genomics and sequencing technology, a number of genes highly associated with PSCC, including DAPK, FHIT, MGMT, CDKN2A (p14ARF), CDKN2A (p16INK4A), RAR-β, and RUNX3, have been identified. They are expected to become important targets for PSCC precision therapy [158]. Second, multiple tumor-associated targets were discovered on the surface of PSCC, which were important targets for ADCs. Finally, studies have shown that EGFR, the human EGFR receptor family, PI3K pathway, JAK-STAT pathway, and BRCA mutations play an important role in PeCa tumor growth and chemotherapy resistance, among which EGFR-targeted drugs are the most widely studied [159].

EGFR has been found to be highly expressed in PeCa. One study reported EGFR overexpression in 91% of cases, and KRAS mutation associated with EGFR resistance was rare [160]. This suggests a potential application of EGFR inhibitors in PeCa. Currently, the most used treatments for PeCa include EGFR monoclonal antibodies (cetuximab, panitumumab, and nimotuzumab) and EGFR TKIs. Based on existing research on EGFR-targeted agents, there is reason to believe that EGFR inhibitors have the potential to become the standard second-line treatment options for PeCa (monotherapy or combination with chemotherapy), but this needs to be confirmed by large-scale prospective clinical studies. In this era of precision oncology, with further elucidation of the molecular pathogenesis of PSCC, more novel and exciting therapeutic methods will continue to emerge.

## Conclusions

With the continuous advancement of science and technology, the treatment of advanced tumors has gradually moved away from traditional methods, such as surgery, radiotherapy, and chemotherapy, and entered the era of immunotherapy and precision therapy [1, 49, 161, 162]. Tumor cells can negatively regulate the body’s immune response through various ways to evade immune surveillance. Tumor immunotherapy aims to restore or enhance the body’s anti-tumor response, inhibiting or eliminating tumor tissue, and combining with targeted ADCs to comprehensively kill tumor tissue from the whole body to the local [9, 17, 51, 52, 141].

Immunotherapy has revolutionized the management of advanced genitourinary tumors. Compared with traditional chemotherapy, immunotherapy has the advantages of better targeting, fewer AEs, higher efficacy, and even the possibility of complete remission for some patients [163]. This makes ICI therapy an important tool for cancer treatment, especially for patients whose first-line therapies have failed.

While there have been many breakthroughs in ICIs for treating RCC and UC, it has been slow progress in PCa and reproductive system tumors. The future development direction is to improve the efficacy of immunotherapy. The ideal ICB therapy should be highly selective and cytotoxic to tumor cells while causing no harm to normal tissues [164]. Therefore, two major strategies for optimizing the efficacy of ICB therapy in the future are to utilize new technologies to more accurately screen patients through biomarkers and take personalized treatment [165]. Different genitourinary tumors have different responses to immunotherapy, which is mainly determined by the IME of the organ where the tumor is located. Altering the IME with new technologies, especially that of PCa, can transform “cold” tumors into immunologically “hot” tumors through combination therapy [105]. This may be an important strategy for addressing low tumor response to immunotherapy in the future. Additionally, although ICIs provide significant survival benefits for some cancer patients, a subset of patients also experience tumor acceleration in the early stage of treatment, known as hyper-progressive disease (HP) [166] HP is a phenomenon of accelerated tumor growth primarily seen in patients with advanced cancer who use ICIs. Patients often experience a serious deterioration in quality of life and a poor prognosis [165, 167]. In the future, how to avoid or delay the occurrence of HP in ICB therapy is a difficult problem that needs to be solved intensively.

**Fig. 3:**
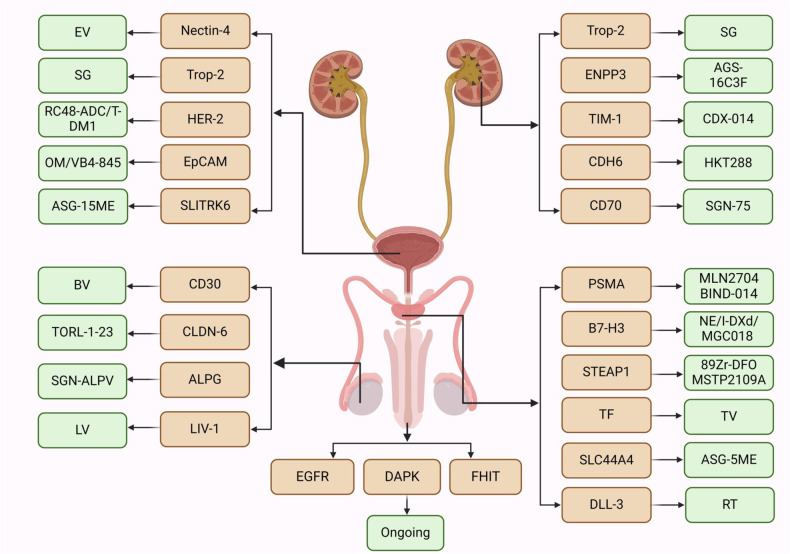
ADCs and their targets in urogenital tumors.

ADCs, as a new type of anti-tumor combination therapy drugs, have great market potential and clinical value. In recent decades, ADCs have made breakthroughs not only in hematological tumors but also in solid tumors [15, 168], especially in urogenital tumors. With the development of biotechnology, an increasing number of tumor-specific antigens have been discovered. These antigens serve as crucial targets for the development of ADCs. As shown in Fig. 3, the relationship between these targets and their corresponding ADCs plays an important role in the treatment of urogenital tumors. Compared with traditional chemotherapy, ADCs have the advantages of low recurrence rate and long-lasting anti-tumor effects [15, 26]. They mobilize the body’s immune system to recognize and destroy tumor cells, thereby reducing the severe side effects of traditional chemotherapy in humans [169].

However, the development and clinical application of ADCs also face many challenges and difficulties, such as insufficient effectiveness, inadequate safety, and narrow therapeutic windows [97, 170, 171]. Due to the difference between antibodies and cytotoxic drugs, the TRAEs spectrum of anti-tumor drugs is also different, which can affect multiple organs in the body [97]. Therefore, patients should be systematically evaluated before receiving ADC therapy. In addition to evaluating routine physical status (ECOG performance status), hematological indicators, and comorbidities, the functional status of the target organs where AEs occur should also be evaluated. For example, if there are rashes, itching, vitiligo, etc., the location and severity should be recorded. When assessing the baseline of cardiovascular and pulmonary function, metabolic and endocrine system status in terms of potential eye disease, blood glucose, and blood lipids should also be evaluated [79, 81, 90, 172].

**Fig. 4:**
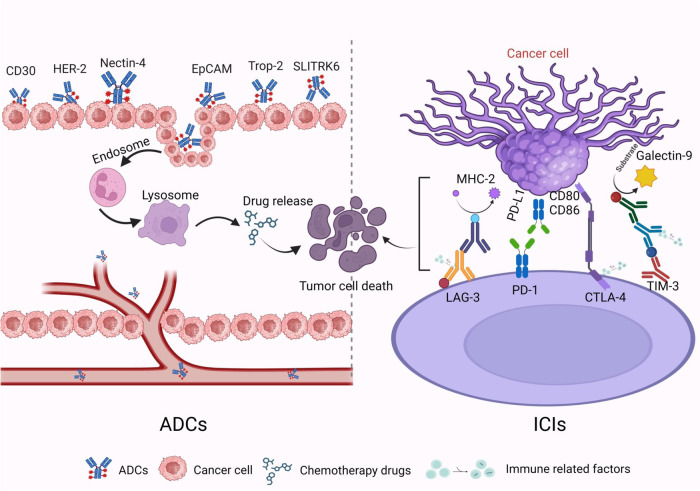
The mechanism of ICIs and ADCs in treating the same tumor, respectively. For ICIs, CTLA-4 competes with CD28 for CD80 and CD86 ligands, thereby inhibiting TCR signaling; PD-1 regulates T cells by interacting with PD-L1; LAG-3 mediates T-cell inhibition through interacting with MHC-2; TIM-3 controls TH1 cell immunity by binding to Galectin-9’s sugar chain and inducing apoptosis. For ADCs, when the drugs enter the bloodstream, they recognize and bind to tumor cells through their antibody components. Drugs entering into tumor cells are wrapped by endosomes, causing lysosomal degradation and the release of cytotoxic drugs, leading to the destruction of tumor cells. Created with BioRender.com.

Multimodal therapy (MMT) has exploratory potential and has gradually become the preferred treatment for advanced urogenital tumors [173]. Current studies have shown that immune-based combination therapies have definite efficacy in first- or second-line treatment of aUC, especially in combination with ADCs [174]. As shown in Fig. 4, the role of ICIs is to alleviate immunosuppression in TME and stimulate the body’s autoimmune response, thus exerting anti-tumor roles. ADCs, on the other hand, combine the targeting abilities of monoclonal antibodies with the cytotoxic properties of anti-tumor chemotherapy drugs through specific biochemical linkers. This approach is called “targeted chemotherapy” and is designed to minimize cytotoxicity. By delivering the drug directly to the tumor, it maximizes its effectiveness. The combination of these two treatments can synergistically eliminate tumor cells. Some scholars believed that ADCs were one of the therapeutic strategies for patients with immunotherapy-resistant and immunologically “cold” tumors [175].

Although MMT has made some achievements, there are still some problems to be solved. Firstly, more basic experiments are needed to elucidate the underlying mechanisms of combination therapy. Secondly, the administration sequence, dosage selection, and AE control of combination therapy are still worthy of attention. Thirdly, reliable biomarkers are needed to predict the anti-tumor effects.

In summary, whether ADCs are used alone or in combination with ICIs, they face some limitations and challenges: (1) The efficacy of ICIs and ADCs is different, and not all patients will respond to treatment with these drugs. There are many reasons for this phenomenon, which requires us to fully consider the individual differences of patients in specific clinical practice. (2) Patients with different health conditions need a more precise and refined dosage to achieve the best clinical effects and the lowest AEs. (3) The high price of drugs for ICIs and ADCs therapy, especially for patients requiring long-term treatment, can impose a large financial burden, which may limit clinical use. (4) The predictive biomarkers of combination therapy strategies are not clear enough, so we can only use drugs based on previous experience. Fortunately, with the continuous development of molecular diagnostics, various biomarkers, such as PD-L1, TMB, molecular subtypes, and lncRNAs, are being studied [176]. Personalized studies utilizing MMT based on multiple biomarkers are also underway and may become a new therapeutic strategy for urogenital tumors. ICIs-ADCs strategy is about to enter the era of precision medicine.

### Supplementary information

Supplementary information
