# Supplementary material for: The combination of immune checkpoint inhibitors and antibody-drug conjugates in the treatment of urogenital tumors: a review insights from phase 2 and 3 studies
Source: Cell Death Dis. 2024 Jun 19;15(6):433. doi: 10.1038/s41419-024-06837-w (PMC11186852; doi:10.1038/s41419-024-06837-w)
Supplement: Supplementary file 1 — Supplementary information [file 41419_2024_6837_MOESM1_ESM.pdf]

Supplementary Table legends

Supplementary Table S1. Ongoing clinical trials in UC.

Supplementary Table S2. Ongoing clinical trials in PCa.

Supplementary Table S3. Ongoing clinical trials in TGCT.

Supplementary Table S4. Ongoing clinical trials in PeCa.

Supplementary Table S1. Ongoing clinical trials in UC.

Categories	Clinical trials	Phases	Patients	Drugs	Targets	Clinical outcomes	References
ICIs	MK-3475-992	3	636 MIBC	Pemb	PD-1	Not published yet.	From:https://clinicaltrials.gov/
ADCs	RC48-C009	2	60 la/mUC	RC48-ADC	HER-2	Not published yet.	From:https://clinicaltrials.gov/
	RC48-C014	2	36 UC	RC48-ADC	HER-2	Not published yet.	From:https://clinicaltrials.gov/
	NCT04073602	2	19 la/mUC	RC48-ADC	HER-2	Not published yet.	From:https://clinicaltrials.gov/
	NCT05016973	2	25 MIBC	RC48-ADC+Trip	HER-2+CD3	Not published yet.	From:https://clinicaltrials.gov/
	IMMU-132-13	3	696 aUC	SG	TROP-2	Not published yet.	From:https://clinicaltrials.gov/
	SURE-01	2	56 MIBC	SG	TROP-2	Not published yet.	From:https://clinicaltrials.gov/
	NCT02449239	3	133 nmBC	OM	EpCAM	Not published yet.	From:https://clinicaltrials.gov/
	NCT04859751	3	53 NMBC	VB4-845	EpCAM	Not published yet.	From:https://clinicaltrials.gov/
ICIs+ADCs	NCT05239624	2	23 BC	Pemb+EV	PD-1+ Nectin-4	Not published yet.	From:https://clinicaltrials.gov/
	NCT03924895	3	857 MIBC	Pemb+EV	PD-1+ Nectin-4	Not published yet.	From:https://clinicaltrials.gov/
	NCT04700124	3	784 MIBC	Pemb+EV	PD-1+ Nectin-4	Not published yet.	From:https://clinicaltrials.gov/
	NCT03606174	2	260 la/mUC	Pemb+EV	PD-1+ Nectin-4	Not published yet.	From:https://clinicaltrials.gov/
	NCT04960709	3	830 MIBC	Durv/Trem+EV	PD-L1+ Nectin-4	Not published yet.	From:https://clinicaltrials.gov/
	NCT03869190	2	645 mUC	Atez+EV	PD-L1+ Nectin-4	Not published yet.	From:https://clinicaltrials.gov/
	NCT04863885	2	46 mBC	Nivo+Ipil+ SG	PD-1+ CTLA-4+TROP-2	Not yet recruiting.	From:https://clinicaltrials.gov/

Supplementary Table S2. Ongoing clinical trials in PCa.

Categories	Clinical trials	Phases	Patients	Drugs	Targets	Clinical outcomes	References
	NCT04145622	2	195 cancers (including PCa)	I-DXd	B7-H3	Not published yet.	From:https://clinicaltrials.gov/
ADCs	NCT03729596	2	143 cancers (includuing mCRPC	MGC018	B7-H3	Not published yet.	From:https://clinicaltrials.gov/
	NCT05551117	2	100 mCRPC	MGC018	B7-H3	Not published yet.	From:https://clinicaltrials.gov/

Supplementary Table S3. Ongoing clinical trials in TGCT.

Categories	Clinical trials	Phases	Patients	Drugs	Targets	Clinical outcomes	References
ICIs+ADCs	NCT06041503	2	68 TGCT	Pemb+BV	PD-1+CD30	Not yet recruiting.	From:https://clinicaltrials.gov/

Supplementary Table S4. Ongoing clinical trials in PeCa.

Categories	Clinical trials	Phases	Patients	Drugs	Targets	Clinical outcome	Reference
ICIs	NCT04224740	2	37 PeCa	Pemb	PD-1	Active, not recruiting.	From:https://clinicaltrials.gov/
	NCT02721732	2	202 PeCa	Pemb	PD-1	Active, not recruiting.	From:https://clinicaltrials.gov/
	NCT03391479	2	24 PeCa	Avel	PD-L1	Active, not recruiting.	From:https://clinicaltrials.gov/
	NCT03686332	2	32 PeCa	Atez	PD-L1	Active, not recruiting.	From:https://clinicaltrials.gov/

15th May., 2024

Language Certificate

Dear Sir or Madam:

This document certifies that the manuscript **MJD2405054**:

The combination of immune checkpoint inhibitors and antibody-drug conjugates in the treatment of urogenital tumors: A review of phase 2 and 3 studies

was edited for proper English language, grammar, punctuation, spelling, and overall style by a highly qualified, native English-speaking professional scientific editor at Medjaden Inc. on 15th May., 2024, Neither the research content nor the authors' intentions were altered in any way during the editing process. The final revised version can be found at http://pan.medjaden.com:2000/index.php?mod=shares&sid=ZWFtREVQYlZ2YXhxREtxQnd5d2h6Y1ZoNUh2VXg1X0VUT3R5aEcyNw(verification code: **2405054**)

Yours sincerely,

(Ms.) Hua (Selin) He for Medjaden Inc.

Information about Medjaden Inc.

Medjaden Inc. is an emerging editing company that provides professional services for laboratory researchers, postgraduates, and clinical doctors. Our mission is to help our clients initiate, maximize and accelerate the production of articles from their research work. The company employs experienced medical statisticians and editors drawn from English backgrounds worldwide, who strive to ensure that every edited article is published in an English journal, preferably one that is included in the Web of Science (WOS), the Science Citation Index (SCI) Expanded, Medline, PubMed, EMBASE, and other scientific resources.

Email: medjaden@gmail.com; Website: www.medjaden.com
